# Incidence and Outcome of Coinfections with SARS-CoV-2 and Rhinovirus

**DOI:** 10.3390/v13122528

**Published:** 2021-12-16

**Authors:** Elisabeth Le Glass, Van Thuan Hoang, Céline Boschi, Laetitia Ninove, Christine Zandotti, Aurélie Boutin, Valérie Bremond, Grégory Dubourg, Stéphane Ranque, Jean-Christophe Lagier, Matthieu Million, Pierre-Edouard Fournier, Michel Drancourt, Philippe Gautret, Philippe Colson

**Affiliations:** 1 Institut Hospitalo-Universitaire Méditerranée Infection, 19-21 Boulevard Jean Moulin, 13385 Marseille, France; elisabeth.le-glass@ap-hm.fr (E.L.G.); celine.boschi@ap-hm.fr (C.B.); laetitia.ninove@ap-hm.fr (L.N.); christine.zandotti@ap-hm.fr (C.Z.); gregory.dubour@ap-hm.fr (G.D.); stephane.ranque@univ-amu.fr (S.R.); jean-christophe.lagier@univ-amu.fr (J.-C.L.); matthieu.million@ap-hm.fr (M.M.); pierre-edouard.fournier@univ-amu.fr (P.-E.F.); michel.drancourt@univ-amu.fr (M.D.); philippe.gautret@ap-hm.fr (P.G.); 2 Thai Binh University of Medicine and Pharmacy, Thai Binh 410000, Vietnam; thuanytb36c@gmail.com; 3 Aix Marseille Univ, IRD, AP-HM, MEPHI, 27 Boulevard Jean Moulin, 13005 Marseille, France; 4 Aix-Marseille Univ, IRD 190, Inserm 1207, Unité des Virus Émergents (UVE), 27 Boulevard Jean Moulin, 13005 Marseille, France; 5 Assistance-Publique-Hôpitaux de Marseille, Service des Urgences Pédiatriques, CHU Timone, 264 rue Saint-Pierre, 13005 Marseille, France; aurelie.boutin@ap-hm.fr (A.B.); valerie.bremond@ap-hm.fr (V.B.); 6 Aix Marseille Univ, IRD, AP-HM, SSA, VITROME, 27 Boulevard Jean Moulin, 13005 Marseille, France

**Keywords:** SARS-CoV-2, COVID-19, rhinovirus, coinfection, severity

## Abstract

Background: We aimed to compare the clinical severity in patients who were coinfected with severe acute respiratory syndrome coronavirus 2 (SARS-CoV-2) and rhinovirus or monoinfected with a single one of these viruses. Methods: The study period ranged from 1 March 2020 to 28 February 2021 (one year). SARS-CoV-2 and other respiratory viruses were identified by real-time reverse-transcription-PCR as part of the routine work at Marseille University hospitals. Bacterial and fungal infections were detected by standard methods. Clinical data were retrospectively collected from medical files. This study was approved by the ethical committee of our institute. Results: A total of 6034/15,157 (40%) tested patients were positive for at least one respiratory virus. Ninety-three (4.3%) SARS-CoV-2-infected patients were coinfected with another respiratory virus, with rhinovirus being the most frequent (62/93, 67%). Patients coinfected with SARS-CoV-2 and rhinovirus were significantly more likely to report a cough than those with SARS-CoV-2 monoinfection (62% vs. 31%; *p* = 0.0008). In addition, they were also significantly more likely to report dyspnea than patients with rhinovirus monoinfection (45% vs. 36%; *p* = 0.02). They were also more likely to be transferred to an intensive care unit and to die than patients with rhinovirus monoinfection (16% vs. 5% and 7% vs. 2%, respectively) but these differences were not statistically significant. Conclusions: A close surveillance and investigation of the co-incidence and interactions of SARS-CoV-2 and other respiratory viruses is needed. The possible higher risk of increased clinical severity in SARS-CoV-2-positive patients coinfected with rhinovirus warrants further large scale studies.

## 1. Introduction

Severe acute respiratory syndrome coronavirus 2 (SARS-CoV-2) emerged in France at the end of January 2020. Since then, on 23 September 2021, the number of cases reached 6,905,071 and 115,517 associated deaths were recorded [1]. In our institute, the first case was diagnosed at the end of February 2020 and, since then (on 31 August 2021), 57,055 patients were found positive among the 525,464 patients tested. We have previously observed that the frequency of coinfections with several respiratory viruses and the type of respiratory viruses involved were a “matter of sampling time” [2]. Indeed, very important differences were reported according to the period of the study and the region of the world where it took place as the occurrence of viral coinfections requires a co-incidence of the viral epidemic periods [2,3,4,5,6]. Likewise, major differences were observed according to the epidemic mode of the respiratory viruses involved in coinfections, i.e., according to whether they showed a bell-shaped curve of incidence or circulated throughout the year with varying intensities [7]. Contrasting with our previous work that focused on the March-April 2020 period, we studied the diagnoses of infections with SARS-CoV-2 and/or other respiratory viruses over a whole year during the SARS-CoV-2 pandemic. We centered our analyses on coinfections with SARS-CoV-2 and human rhinovirus (HRV) as this latter virus is the one that was found the most frequently associated with SARS-CoV-2 (in 41% of detected coinfections) in our center [2]. This predominance has also been reproducibly observed in several other previous studies conducted in various settings and geographical areas, with HRV being reported as the most frequent or among the most frequent respiratory viruses coinfecting COVID-19 patients [2,5,6,8,9,10,11,12,13]. Nonetheless, the clinical outcome of SARS-CoV-2 infection in patients co-infected with HRV remains unknown. In addition, HRV has a less marked seasonality compared to other respiratory viruses as it circulates throughout the year, although with various levels of incidence [7]. This is of particular interest in the study of respiratory virus coinfections with respect to the current SARS-CoV-2 pandemic. Here, we compared clinical severity in patients coinfected with a SARS-CoV-2 and HRV or monoinfected with a single of these viruses.

## 2. Materials and Methods

The study period ranged from 1 March 2020 to 28 February 2021 (one year). Data included the results of all diagnosis tests of infections with SARS-CoV-2 and other respiratory viruses performed as part of routine work at the clinical virology laboratory of IHU Méditerranée Infection and Assistance Publique-Hôpitaux de Marseille (Marseille university hospitals), Marseille, France. The study was approved by the ethical committee of the University Hospital Institute Méditerranée Infection (N°: 2020-029).

SARS-CoV-2 infections were diagnosed on nasopharyngeal samples collected from patients tested by real-time reverse-transcription-PCR (qPCR) using various reagents and protocols, including one previously described [14], as well as the BGI real-time fluorescent RT-PCR (BGI Genomics, Shanghai Fosun Long March Medical Science Co., Ltd., Shenzhen, China), the Biofire FilmArray Respiratory panel 2 plus (Biomérieux, Marcy-l’Etoile, France), the GeneXpert Xpert Xpress SARS-CoV-2 (Cepheid, Sunnyvale, CA, USA), or the VitaPCR SARS-CoV-2 (Credo Diagnostics Biomedical, Singapore) assays. Other viral respiratory infections were diagnosed using the FTD Respiratory pathogens 21 (Fast Track Diagnosis, Luxembourg), the Biofire FilmArray Respiratory panel 2 plus, the Respiratory Multi-Well System r-gene (Argene, bioMérieux, Marcy l’Etoile, France), or the GeneXpert Xpert Flu/RSV (Cepheid, Sunnyvale, CA, USA) assays. Co-infections were assessed on the same respiratory sample. The presence of bacterial and/or fungal pathogens was documented by conventional culture methods coupled with a MALDI-TOF mass spectrometry identification [15,16,17].

For patients coinfected with SARS-CoV-2 and HRV, demographic and clinical data were retrospectively retrieved when they were available from medical files, including comorbidities, main symptoms, in/outpatient status, transfer to an intensive care unit (ICU), and death. To evaluate the relationship between SARS-CoV-2-HRV coinfections and the clinical profiles and outcomes of the patients, we selected patients monoinfected either with SARS-CoV-2 or with HRV as controls groups, matched with cases by, in order of priority, gender, age, comorbidities, and date of diagnosis. Infections with SARS-CoV-2 and/or another respiratory virus diagnosed during the period from 1 March to 30 April 2020 for 1711 patients from the present study were described in a previous work that only focused on diagnoses performed in March and April 2020 [2].

Statistical analyses were carried out using R 4.0.2 (https://cran.r-project.org/, accessed on 1 June 2021) and Stata version 15.1 (http://www.stata.com, accessed on 1 June 2021). Qualitative variables were presented by percentage. The univariable and multivariable analyses were conducted to evaluate the epidemiological and clinical profiles of patients coinfected with SARS-CoV-2 and HRV compared to monoinfected patients. Unadjusted associations between multiple factors (socio-demographic characteristic, comorbidities, and clinical profiles) and SARS-CoV-2 infection with or without HRV coinfection were investigated. Multivariable analysis was performed using logistic regression. Variables with a *p*-value < 0.2 in the univariate analysis were included in the multivariate analysis. The results were presented by an odd ratio (OR) with a 95% confidence interval (CI). A *p*-value < 0.05 was considered statistically significant.

## 3. Results

**Figure 1:**
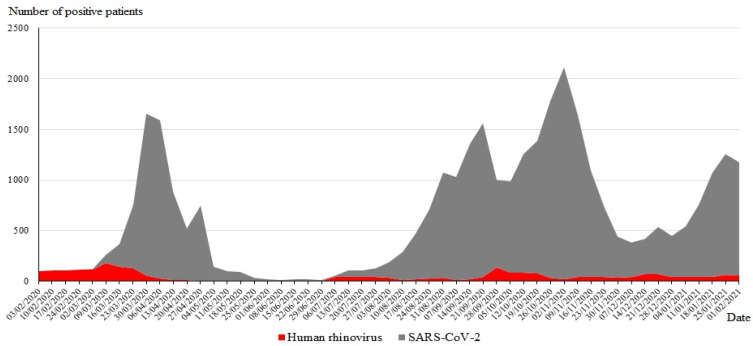
Temporal distribution of the SARS-CoV-2 and HRV diagnoses from 1 March 2020 to 28 February 2021.

During the study, 15,157 patients were tested for SARS-CoV-2 and other respiratory viruses, with 6034 (40%) testing positive for at least one virus. The distribution of infections with respiratory viruses, including SARS-CoV-2, diagnosed from 1 March 2020 to 28 February 2021 (one year), is shown on Figure 1. Ninety-three (4.3%) SARS-CoV-2-infected patients were coinfected with another virus, with HRV being the most frequent (*n* = 62/93; 67%), followed by adenoviruses (14; 15%) (Table 1). Among the 3875 SARS-CoV-2-negative patients, HRV was also the virus the most frequently detected in 2337 cases (39%), followed by adenoviruses (446; 7%).

We aimed to compare the clinical severity and outcome of coinfections with SARS-CoV-2 and HRV and of monoinfections with one of these two viruses. Four of the sixty-two SARS-CoV-2-HRV-coinfected patients (7%) were excluded due to the lack of clinical data, including two males and two females with ages of 9, 12, 25, and 26 years. Using a randomly matched selection based on gender and age, 58 patients with SARS-CoV-2 monoinfection and 58 patients with a HRV monoinfection were selected as controls (Table 2); 43% of these patients were children under 15 years of age and 57% were male.

Concurrent infections with bacteria or microscopic fungi were found in ten patients. Three patients coinfected with SARS-CoV-2 and HRV were also coinfected with *Candida albicans* in one case, *Stenotrophomonas maltophilia* in one case, and with multiple bacterial and fungal pathogens (*Haemophilus influenzae*, *Pseudomonas aeruginosa*, *Enterobacter gergoviae*, and *Candida lusitaniae*) in one case. Three and four other patients in the SARS-CoV-2 and HRV monoinfection groups, respectively, were also coinfected with bacteria (Supplementary Table S1).

Table 3 shows the univariate and multivariate analyses comparing the clinical profiles and outcomes of the three groups of patients. In the univariate analysis, patients coinfected with SARS-CoV-2 and HRV were significantly more likely to report a cough than those with SARS-CoV-2 monoinfection (62% versus 31%; *p* = 0.0008). They were also significantly more likely to report dyspnea than patients with HRV monoinfection (45% versus 36%; *p* = 0.02). These associations remained significant in the multivariate analysis. In addition, patients who were coinfected with SARS-CoV-2 and HRV had a non-statistically significant increased risk of being transferred to an intensive care unit (ICU) and to die than those infected only with HRV (16% versus 5% and 6.9% versus 1.7%, respectively).

Finally, ten patients with SARS-CoV-2 and/or HRV infections were also diagnosed with bacteria or *Candida* spp. The detection of these microorganisms can indicate the most likely colonizations rather than bronchopulmonary infections. Of these ten patients, seven were hospitalized (two coinfected with SARS-CoV-2 and rhinovirus, three SARS-CoV-2-monoinfected, and two rhinovirus-monoinfected), three were transferred to an ICU (two SARS-CoV-2 and rhinovirus-coinfected and one SARS-CoV-2-monoinfected), and two died (one SARS-CoV-2 and rhinovirus-coinfected and one SARS-CoV-2-monoinfected) (Supplementary Table S1).

## 4. Discussion

Over a whole year from March 2020 through February 2021, rhinovirus was, by far, the most frequently diagnosed respiratory virus in our institute, either in association with SARS-CoV-2 or alone. To our knowledge, to date, no study systematically assessed the severity of infections with SARS-CoV-2 and rhinovirus, but prolonged SARS-CoV-2 persistence beyond the acute infection phase was significantly associated with HRV/enterovirus co-infection [11]. It could increase the capacity of viral transmission [11]. Previous reports were case reports [18,19]. Orozoco-Hermandez et al. reported increased severity of initially mild COVID-19 symptomatology in a 41-year-old patient with coinfection with rhinovirus or enterovirus who developed multilobar pneumonia requiring admission to ICU [18]. A case of SARS-CoV-2 and rhinovirus–enterovirus coinfection was also reported in a pregnant woman [19]. Our results notably suggest that patients coinfected with SARS-CoV-2 and rhinovirus were more likely to suffer dyspnea than those infected with rhinovirus only. A trend toward increased risks for both transfer to an ICU and death was also seen in patients coinfected with SARS-CoV-2 and rhinovirus compared to those monoinfected with rhinovirus. The putative interactions and clinical impact of HRV and SARS-CoV-2 co-infections were, thus, very scarcely addressed. Here, co-infected patients presented significantly more frequently cough, compared to those with mono-SARS-CoV-2 infection. Although the interaction between these viruses is still unclear, the co-detection of HRV has also been associated with mild COVID-19 [10,12]. One potential reason for this phenomenon is that viral coinfection is more common in young persons, aged between 15 and 64 years [10]. Besides, a recent in vitro study reported an indirect negative interaction between HRV and SARS-CoV-2 and hypothesized that HRV can trigger an interferon response that makes most cells nonpermissive to SARS-CoV-2 infection, blocking its replication [20].

The present study has some limitations. Not all patients diagnosed as infected with SARS-CoV-2 or HRV during the study period were systematically tested for the other respiratory virus. In addition, we were not able to classify these cases as concomitant infections or superinfections, as the study was not designed for this aim and same respiratory samples were tested for both viruses that have different incubation duration and may differ regarding their persistence duration. In addition, several biomarkers known to be associated with respiratory disease severity, including lactate dehydrogenase level, D-dimers value, thrombocyte count, and troponin level [21], were not considered either. Moreover, we did not provide information on the duration of symptoms which could differ between patients who were co- or monoinfected with SARS-CoV-2 and rhinovirus. Additionally, we did not follow up the patient after discharge. Finally, the duration and intensity of viral shedding were not evaluated. Since the future of the current SARS-CoV-2 pandemic is unknown, a close surveillance and investigation of the co-incidence and interactions of SARS-CoV-2 and other respiratory viruses are needed. Further large-scale studies are needed to investigate the role of co-infection between SARS-CoV-2 and HRV in severity of COVID-19.

## Figures and Tables

**Table 1:** Epidemiological and virological features of SARS-CoV-2-negative and -positive patients coinfected with other respiratory viruses using the FTD Respiratory pathogens 21 (Fast Track Diagnosis, Luxembourg), the Biofire FilmArray Respiratory panel 2 plus (Biomérieux, Marcy-l’Etoile, France), the Respiratory Multi-Well System r-gene (Argene, BioMérieux), or the GeneXpert Xpert Flu/RSV (Cepheid, Sunnyvale, CA, USA) assays.

Epidemiological Features and Viruses	All Patients (*n* = 6034)	SARS-CoV-2-Negative but Positive for Another Respiratory Virus * (*n* = 3875)	SARS-CoV-2-Positive without Coinfection (*n* = 2066) **	SARS-CoV-2-Positive with Coinfection (*n* = 93) **
Age (mean ± standard deviation), years	38.1 ± 25.6	35.9 ± 24.9	47.2 ± 23.2	44.5 ± 22.8
Gender				
Male	3249 (53.8)	1982 (51.1)	1218 (59.0)	48 (51.6)
Female	2788 (46.2)	1893 (48.9)	848 (41.1)	45 (48.4)
Influenza viruses				
Influenza A virus	212 (3.5)	210 (3.5)	-	2 (0.1)
Influenza B virus	235 (3.9)	233 (3.9)	-	2 (0.1)
Parainfluenza viruses				
Parainfluenza virus 1	3 (0.05)	3 (0.1)	-	0 (0)
Parainfluenza virus 2	15 (0.3)	12 (0.2)	-	2 (0.1)
Parainfluenza virus 3	360 (6.0)	356 (5.9)	-	4 (0.2)
Parainfluenza virus 4	41 (0.7)	38 (0.6)	-	3 (0.1)
Human coronaviruses				
Coronavirus 229E	42 (0.7)	41 (0.7)	-	1 (0.05)
Coronavirus OC43	173 (2.9)	171 (2.8)	-	2 (0.1)
Coronavirus NL63	268 (4.4)	267 (4.4)	-	1 (0.05)
Coronavirus HKU1	72 (1.2)	70 (1.2)	-	2 (0.1)
SARS-CoV-2	2159 (37.8)	0	2066 (95.7)	-
Respiratory Syncytial virus	46 (0.7)	45 (0.7)	-	1 (0.05)
Bocavirus	95 (1.6)	92 (1.5)	-	3 (0.1)
Adenovirus	446 (7.4)	432 (7.2)	-	14 (0.6)
Metapneumovirus	116 (1.9)	113 (1.9)	-	3 (0.1)
Rhinovirus	2337 (38.7)	2275 (37.7)	-	62 (2.9)
Enterovirus	57 (0.9)	53 (0.9)	-	4 (0.2)

* Proportion of respiratory viruses other than SARS-CoV-2 was calculated in all patients. ** Proportion of respiratory viruses other than SARS-CoV-2 was calculated in 2159 SARS-CoV-2 positive patients.

**Table 2:** Descriptive analysis of SARS-CoV-2 coinfected patients and controls.

Variables	SARS-CoV-2+Rhinovirus Coinfection (*n* = 58)	SARS-CoV-2 Monoinfection (*n* = 58)	Rhinovirus Monoinfection (*n* = 58)	*p*-Value
Age groups				
<15 years	25 (43.1)	25 (43.1)	25 (43.1)	1.0
15–45 years	14 (24.1)	14 (24.1)	14 (24.1)	
45–65 years	9 (15.5)	9 (15.5)	9 (15.5)	
≥65 years	10 (17.3)	10 (17.3)	10 (17.3)	
Gender				
Male	33 (56.9)	33 (56.9)	33 (56.9)	1.0
Female	25 (43.1)	25 (43.1)	25 (43.1)	

**Table 3:** Coinfection with human rhinovirus and severity of COVID-19 diseases.

	SARS-CoV-2 and Rhinovirus Coinfection	SARS-CoV-2 Monoinfection	Rhinovirus Monoinfection	Coinfection versus SARS-CoV-2 Monoinfection		Coinfection versus Rhinovirus Monoinfection	
				Univariate Analysis	Multivariate Analysis	Univariate Analysis	Multivariate Analysis
	*n* (%)	*n* (%)	*n* (%)	OR [95%CI] *p*-Value	Adjusted OR [95%CI] *p*-Value	OR [95%CI] *p*-Value	Adjusted OR [95%CI] *p*-Value
Comorbidities							
Hypertension	8 (13.8)	7 (12.1)	5 (8.6)	1.17 [0.34–4.08] 0.78	-	1.70 [0.45–7.02] 0.38	-
Diabetes	4 (6.9)	6 (10.3)	6 (10.3)	0.64 [0.13–2.90] 0.51	-	0.64 [0.13–2.90] 0.51	-
Cancer	4 (6.9)	1 (1.7)	3 (5.2)	4.22 [0.40–211.48] 0.17	5.26 [0.53–52.00] 0.16	1.36 [0.22–9.68] 0.70	-
Chronic respiratory diseases	12 (20.7)	7 (12.1)	12 (20.7)	1.90 [0.62–6.18] 0.21	-	1.00 [0.37–2.72] 1.00	-
Chronic heart diseases	6 (10.3)	4 (6.9)	5 (8.6)	1.56 [0.35–7.92] 0.51	-	1.22 [0.29–5.39] 0.75	-
Obesity	2 (3.5)	5 (8.6)	2 (3.5)	0.38 [0.03–2.45] 0.24	-	1.00 [0.07–14.24] 1.00	-
Clinical symptoms							
Fever	34 (58.6)	38 (65.5)	29 (50.0)	0.75 [0.33–1.69] 0.44	-	1.42 [0.64–3.15] 0.35	-
Cough	36 (62.1)	18 (31.0)	27 (46.6)	3.64 [1.58–8.45] 0.0008	3.79 [1.74–8.27] 0.001	1.88 [0.84–4.21] 0.09	1.59 [0.74–3.44] 0.24
Dyspnea	26 (44.8)	21 (36.2)	14 (24.1)	1.43 [0.64–3.23] 0.34	-	2.55 [1.08–6.14] 0.02	2.55 [1.15–5.65] 0.02
Rhinitis	16 (27.6)	11 (19.0)	20 (34.5)	1.63 [0.63–4.34] 0.27	-	0.72 [0.30–1.72] 0.42	-
Anosmia ^1,2^	2 (6.1)	5 (15.2)	-	0.36 [0.03–2.46] 0.23	-	-	-
Ageusia ^1,2^	2 (6.1)	4 (12.1)	-	0.47 [0.04–3.59] 0.39	-	-	-
Hypoxemia	19 (32.8)	20 (34.5)	14 (24.1)	0.93 [0.40–2.15] 0.84	-	1.53 [0.63–3.77] 0.30	-
Clinical outcomes							
Hospitalization	25 (43.1)	28 (48.3)	30 (51.7)	0.81 [0.37–1.80] 0.58	-	0.71 [0.32–1.57] 0.35	-
Transfer to an ICU	9 (15.5)	13 (22.4)	3 (5.2)	0.64 [0.22–1.79] 0.34	-	3.37 [0.77–20.22] 0.07	2.42 [0.59–9.96] 0.22
Death	4 (6.9)	3 (5.2)	1 (1.7)	1.36 [0.22–9.68] 0.70	-	4.22 [0.40–211.48] 0.17	2.67 [0.24–29.73] 0.42

^1^ Data on anosmia and ageusia were available in 33 adult patients. ^2^ Variables were not included in univariate and multivariate of coinfection versus rhinovirus monoinfection; ICU, intensive care unit.

## Data Availability

The data that support the findings of this study will be available from [PC] upon reasonable request.

## Supplementary Materials

The following are available online at https://www.mdpi.com/article/10.3390/v13122528/s1, Table S1: Concurrent infections with bacteria or microscopic fungi among studied patients.
