# Supplementary material for: Incidence and Outcome of Coinfections with SARS-CoV-2 and Rhinovirus
Source: Viruses. 2021 Dec 16;13(12):2528. doi: 10.3390/v13122528 (PMC8709236; doi:10.3390/v13122528)
Supplement: Supplementary file 1 [file viruses-13-02528-s001.zip › viruses-1426957-supplementary.pdf]

Supplementary Material to Article:

Incidence and outcome of coinfections with SARS-CoV-2 and rhinovirus

Elisabeth LE GLASS[1 ¥], Van Thuan HOANG[1,2,3 ¥], Céline BOSCHI[1,4], Laetitia NINOVE[1,5], Christine ZANDOTTI[1], Aurélie BOUTIN[6], Valérie BREMOND[6], Grégory DUBOURG[1,4], Stéphane RANQUE[1,2], Jean-Christophe LAGIER[1,4], Matthieu MILLION[1,4], Pierre-Edouard FOURNIER[1,4], Michel DRANCOURT[1,4], Philippe GAUTRET[1,2], Philippe COLSON[1,4] *

1 IHU Méditerranée Infection, 19-21 Boulevard Jean Moulin, 13005 Marseille, France; elisabeth.le-glass@ap-hm.fr (E.L.); thuanytb36c@gmail.com (V.T.H.) ; celine.boschi@ap-hm.fr (C.B.) ; laetitia.ninove@ap-hm.fr (L.N.) ; christine.zandotti@ap-hm.fr (C.Z.) ; gregory.dubour@ap-hm.fr (G.D.) ; stephane.ranque@univ-amu.fr (S.R.) ; jean-christophe.lagier@univ-amu.fr (J.-C.L.) ; matthieu.million@ap-hm.fr (M.M.); michel.drancourt@univ-amu.fr (M.D.); philippe.gautret@ap-hm.fr (P.G.); philippe.colson@univ-amu.fr (P.C.)

2 Aix Marseille Univ, IRD, AP-HM, SSA, VITROME, 27 Boulevard Jean Moulin, 13005 Marseille, France

3 Thai Binh University of Medicine and Pharmacy, P. Kỳ Bá, Thái Bình, Thai Binh, Viêt Nam

4 Aix Marseille Univ, IRD, AP-HM, MEPHI, 27 Boulevard Jean Moulin, 13005 Marseille, France

5 Aix-Marseille Univ, IRD 190, Inserm 1207, Unité des Virus Émergents (UVE), 27 Boulevard Jean Moulin, 13005 Marseille, France

6 Assistance-Publique-Hôpitaux de Marseille, service des urgences pédiatriques, CHU Timone, 264 rue Saint-Pierre, 13005 Marseille, France; aurelie.boutin@ap-hm.fr (A.B.); valerie.bremond@ap-hm.fr (V.B.)

¥ Contributed equally

* Correspondence: Pr. Philippe Colson, Institut Hospitalo-Universitaire Méditerranée Infection, 19-21 Boulevard Jean Moulin 13385 Marseille Cedex 05, France. Phone: + 33 (0) 4 13 73 24 01. Fax: + 33 (0) 4 13 73 24 02. E-mail address: philippe.colson@univ-amu.fr

Supplementary Table

Supplementary Table S1. Concurrent infections with bacteria or microscopic fungi among studied patients.

Patient	Group	Concurrent infection with other pathogens	Hospitalization	Transfer to ICU	Death
1	SARS-CoV-2 and rhinovirus coinfection	*Haemophilus influenzae, Pseudomonas aeruginosa, Enterobacter gergoviae* and *Candida lusitaniae*	No	No	No
2	SARS-CoV-2 and rhinovirus coinfection	*Candida albicans*	Yes	Yes	No
3	SARS-CoV-2 and rhinovirus coinfection	*Stenotrophomonas maltophilia*	Yes	Yes	Yes
4	SARS-CoV-2 monoinfection	*Mycoplasma pneumoniae*	Yes	No	No
5	SARS-CoV-2 monoinfection	*Moraxella catarrhalis*	Yes	No	No
6	SARS-CoV-2 monoinfection	*Staphylococcus epidermidis*	Yes	Yes	Yes
7	Rhinovirus monoinfection	*Streptococcus pneumoniae*	Yes	No	No
8	Rhinovirus monoinfection	*Klebsiella pneumoniae*	No	No	No
9	Rhinovirus monoinfection	*Mycoplasma pneumoniae*	No	No	No
10	Rhinovirus monoinfection	*Pseudomonas aeruginosa*	Yes	No	No
